# Emotion recognition of social media users based on deep learning

**DOI:** 10.7717/peerj-cs.1414

**Published:** 2023-06-14

**Authors:** Chen Li, Fanfan Li

**Affiliations:** 1 Institute of Arts and Humanities, Shanghai Jiao Tong University, Shanghai, China; 2 Student Affairs Department, Huanggang Normal University, Huanggang, Hubei, China

**Keywords:** Deep learning, Machine learning, Social media, Sentiment characteristics, Emotional analysis

## Abstract

Issues with sentiment analysis in social media include neglecting the long-distance semantic link of emotional features, failing to capture the feature words with emotional hue effectively, and depending excessively on manual annotation. This research provides a user emotion recognition model to achieve the emotional analysis of microblog public opinion events. Three types of inspiring text, “joy,” “anger,” and “sadness,” are obtained by the data collecting and data preprocessing of micro-blog public opinion event comment text. Then, an algorithm using the linear discriminant analysis (LDA) model, emotion dictionary, and manual annotation is created to extract emotional feature words. The captured motivational text is converted into a word vector using Word2vec. After gathering the long-distance semantic data with bidirectional long short-term memories (BiLSTM) and convolutional neural networks (CNN) extract the text’s key characteristics to finish the emotion categorization. The test results demonstrate an average increase in F1 value of 3.66 percent for six machine learning models and an average increase in F1 value of 1.84 percent for seven deep learning models. The suggested model performs better at identifying the emotions of social media users than the current machine learning and deep learning methods.

## Introduction

With the rapid proliferation of Web 2.0 and social media, the internet has become a treasure trove of comment information, containing users’ value tendencies and emotional coloring on public opinion events, character views, and scenery. This information reflects the public’s emotions and attitudes towards various phenomena, including joy, anger, sadness, approval, and criticism. The automatic and expeditious extraction of users’ emotional tendencies from unstructured comments is crucial for dynamically monitoring the emotional state of public opinion events. Hence, the advent of sentinel analysis is a much-needed development (Chen, Jin & Lin, 2021). Sentiment analysis, also known as opinion mining and tendency analysis, aims to analyze, process, reason, and predict subjective texts with emotional coloring, focusing on the distinguishing features of different emotional hues, such as positivity or negativity. Moreover, personal emotion, an important concept closely related to sentiment, plays a significant role (Yang et al., 2020). According to Liu (2010), an emotional sentence expresses personal feelings, opinions, or beliefs, while an objective sentence lacks emotion.

**Figure 1:**
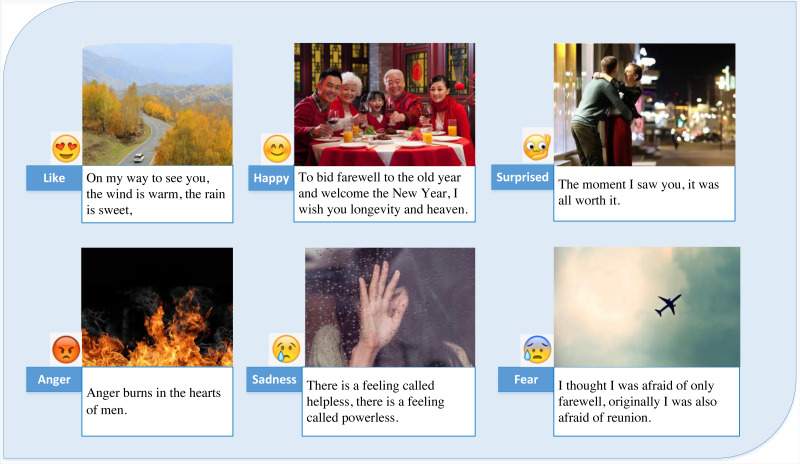
Weibo users’ emotional expression. Content source: https://weibo.com. In expressing feelings, judgments, appreciation, speculation, and recognition, subjective sentences to a certain extent will involve emotions. In addition, emotions can be visible as the declaration of one’s emotions and contemplations. The concept of emotion is close to emotion. Opinion intensity is related to some emotional intensity, such as happiness, surprise, anger, sadness or fear, as shown in the figure.

Furthermore, subjective phrases inherently include emotions to some degree when expressing feelings, assessments, appreciations, hypotheses, and recognitions. Disclosing one’s feelings and thoughts is another way to express emotions. Subjective emotion and the idea of emotion are connected. As seen in Fig. 1, the intensity of an opinion is frequently linked to the intensity of an emotion, such as joy, surprise, wrath, sadness, or fear.

The emergence of social media has made it easier for individuals to share their information. Government and businesses must communicate their sentiments and firsthand knowledge of the occurrences. Bollen, Mao & Pepe (2011) conducted a six-dimensional analysis of public emotions using the profile of mood states. Another study examined the emotional shifts experienced by Twitter users following key events using Prazick’s theory of emotional development psychology to map eight emotions into four pairs of emotional polarities (Wang, Wang & Feng, 2016). However, social media generates varying opinion data because of its large user base. Most of the time, the information is concise and packed with many remarks and personal feelings. There is a lot of text noise, which somewhat enhances the complexity of text mining research (Liu, Qi & Xu , 2021). Traditional sentiment analysis approaches extract characteristics from text data to achieve sentiment classification. The results are somewhat biased since they do not adequately consider the semantic importance between contexts, which cannot reflect the true feelings of social media.

The previous social media emotion analysis ignored the long-distance semantic relationship of emotion features. It could not accurately capture the feature words with emotional color in text information, so a large number of manual labeling was needed to improve the experimental results. Therefore, this article proposes a user emotion recognition model. The main contributions are as follows:

• A model integrating the LDA model, emotion dictionary and manual annotation is constructed and used for emotion feature word extraction. Word2Vec is used to convert the emotion text after feature extraction into word vector to construct an emotion word database for microblog social media;

• The CNN-BiLSTM model is constructed. CNN is used to extract the key features of text, and BiLSTM captures the long-distance semantic features. Finally, Softmax classifiers are used to calculate the emotional tendency of comments in social media public opinion events.

The LDA model and emotion dictionary are integrated to achieve feature extraction. Then, the CNN-BiLSTM model is constructed to complete the sentiment classification to predict the emotional situation of public opinion events and realize the emotional analysis of users to a certain extent, which has specific research significance.

## Related Works

Relying on social media, users have generated many opinions on public opinion events, people’s views and scenery, which provides the possibility to understand and deeply mine the user’s information behavior. Its core research is to analyze the emotions that users express on the social media platform, that is, emotional analysis. The emotional analysis mainly includes orientation classification, sentiment analysis, emotion time series analysis, subjective detection, opinion summary, opinion retrieval, opinion holder extraction, irony and irony detection, cross-domain sentiment analysis and multimodal sentiment analysis (Zhao, 2021). The most common sentiment analysis is emotion classification and sentiment analysis. Emotion classification is based on the assumption that an entity or its aspects and attributes can be divided into two opposite emotional polarity, which can be divided into positive, negative and neutral. Mood analysis is based on emotional analysis and combined with the profile of mood states (Li, Lin & Lin, 2018). Kramer, Guillory & Hancock (2014) proposed that emotions on social media platforms can be transmitted through the emotional contagion mechanism based on the experimental research of users of the Facebook platform. The study found that people in the social network environment will unconsciously experience the same emotional state as their friends. Yu & Wang (2015) analyzed the Twitter data during the 2014 World Cup and found that the emotion of users’ tweeting is consistent with the actual situation on the field.

Depending on the feature set, machine learning-based sentiment analysis methods can be divided into supervised learning technology and unsupervised learning technology. Based on the supervised machine learning method, support vector machine (SVM), naive Bayes, decision tree algorithm, *etc*., which needs sufficient corpus as support (Wang et al., 2021). Based on unsupervised learning, the primary methods are unsupervised and semi-supervised learning. To a certain extent, it can solve the analysis limitations brought about by the lack of a complete tagging corpus. Because of the limitations of the machine learning algorithm, scholars optimized the algorithm to a certain extent to improve its recognition effect. Che & Li (2021) integrated an emotion dictionary based on a traditional machine-learning algorithm to improve the recognition effect. Based on the conventional LDA model, Wang & Hu (2020) combined the intuitionistic fuzzy TOPSIS method to calculate the comprehensive evaluation value of the online reviews of agricultural products and effectively found a positive correlation between the comprehensive evaluation value and the positive emotional value. Due to the large number of personal emotions in the corpus of social media and the noise of corpus information, the machine learning method cannot accurately predict emotional features.

Compared with the machine learning method, the deep learning model is no longer dependent on feature extraction but on autonomous learning. With the deepening of deep learning research, the accuracy of the sentiment analysis method based on deep learning gradually exceeds that of traditional methods. Although a deep learning model for sentiment analysis can effectively solve the problem of corpus tagging and has a high accuracy, model training takes a lot of time and cannot explain the final semantics. Given the limitations of the deep learning model at the present stage, scholars have improved the framework model to a certain extent. Xia, Yang & Xue (2021) proposed a self-attention bidirectional hierarchical semantic model for sentiment analysis of web documents, which improved the speed and accuracy of the deep learning model. Zhang, Wang & Deng (2021) used “cold start” to make automatic indexing for ancient poetry texts for corpus learning and adopted the deep learning model BERT-BiLSTM-CRF to conduct emotional analysis of long poems and articles, which effectively improved the accuracy and widens the semantic analysis of intangible cultural heritage texts. Because the message text of social media is short and compact, the deep learning method should consider the semantic relevance of the text before and after the corpus learning and then conduct an emotional analysis.

## Social Media User Emotion Recognition Model Based on Deep Learning

To better analyze the comments of social media users, this article proposes an improved user emotion recognition model, which integrates the LDA model and emotion dictionary to achieve feature extraction. Then, it constructs the CNN-BiLSTM model to complete sentiment classification and predict the emotional situation of public opinion events.

### Overall framework

**Figure 2:**
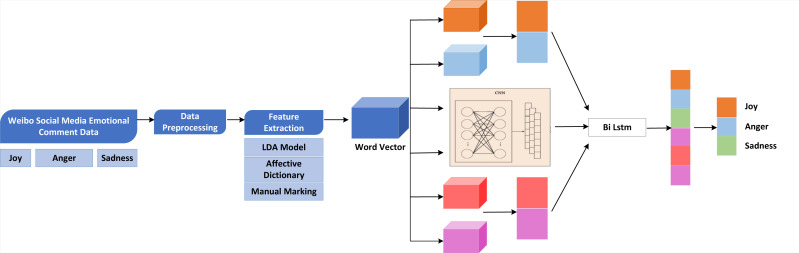
Overall framework of the model. This article proposes an improved LDA and CNN-BiLSTM emotion classification model. The overall framework is shown.

This article proposes an improved LDA and CNN-BiLSTM emotion classification model. The overall framework is shown in Fig. 2 (Rhanoui et al., 2019; Liu et al., 2020).

The specific implementation process is as follows:

(1) Through Python and XPath technology, the user-defined web crawler is used to collect the comment information of microblog social media public opinion events, including “Joy,” “anger,” and “sadness,” and store them in the local CSV file.
(2) The data preprocessing of comment text includes Jieba Chinese word segmentation, stop word filtering, special character deletion, duplicate comment deletion, comment annotation, *etc*.
(3) The deep features of the word model are extracted from the model and used as the input feature of the word model.
(4) The CNN-BiLSTM model is constructed, and CNN is used to extract the key features of the text, and LSTM captures the long-distance semantic features. Finally, the Softmax classifier calculates the sentiment tendency of social media public opinion event comments to complete the emotion classification. The output results correspond to “joy,” “anger,” and “sadness,” respectively.

### Emotional feature extraction

**Figure 3:**
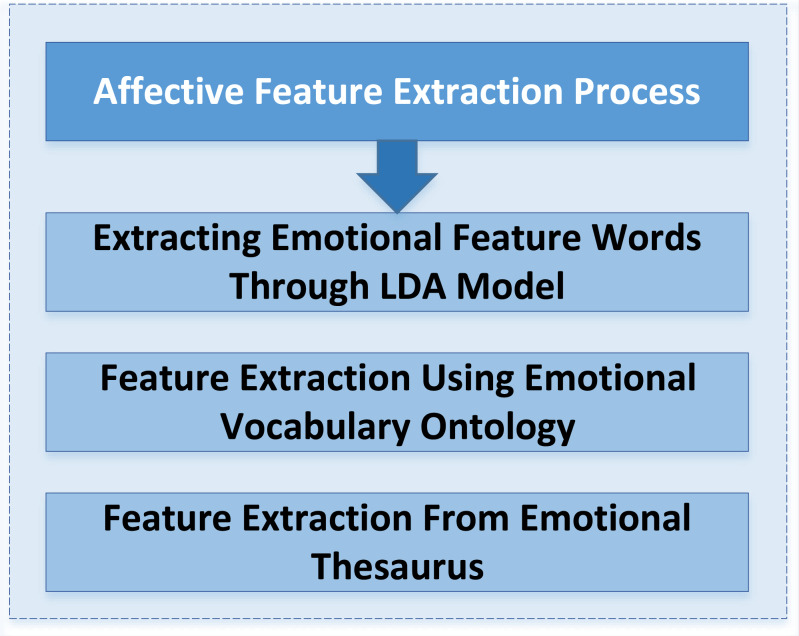
Process of emotional feature extraction. A feature extraction method based on the LDA model and emotion dictionary is constructed. The specific implementation process is shown.

A feature extraction method is constructed using the LDA model and emotion dictionary. Figure 3 shows its specific implementation process.

(1) The LDA model is used to extract the emotional feature words of different review texts, a topic model proposed by Blei & Jordan (2003).
(2) Feature extraction is carried out by using an emotional vocabulary ontology database. At the same time, combined with the word frequency statistics and manual tagging of inspirational feature words, the emotional lexicon for microblog social media is constructed.
(3) The feature extraction task is completed by the emotional lexicon. This operation can extract high-quality feature words with emotional color in different reviews and support the subsequent deep learning model to implement emotion classification tasks.

### CNN model

A convolutional neural network (CNN) mainly comprises convolution and pooling layers. In this article, a three-layer CNN is constructed to extract the key features of comment text with sentiment events. The convolution layer will receive the emotion feature matrix of *n* × *d*, and the convolution process is shown in Eq. (1).

(1) \begin{eqnarray*}{h}_{i}^{d}=f \left( {w}_{d}\times {V}_{i}+{b}_{d} \right) \end{eqnarray*}

In the formula, *f* represents the activation function, and the ReLu function is usually used to accelerate the training convergence speed. *h*^*d*^ represents the feature of comments on Weibo social media after vector convolution processing; *w*_*d*_ represents the convolution kernel of size *d*; *V*_*i*_ represents the word vector of the input layer. *b*_*d*_ represents the offset item. This convolution operation can effectively generate local feature sets, as shown in Eq. (2).

(2) \begin{eqnarray*}{H}_{d}= \left\{ {h}_{1}^{d},{h}_{2}^{d},\ldots ,{h}_{n-d+1}^{d} \right\} .\end{eqnarray*}

The pooling layer can compress the size of text feature vectors and model parameters, maximizing emotional feature retention. Its calculation formula is shown in Eq. (3).

(3) \begin{eqnarray*}{s}_{i}=\max \nolimits \left\{ {H}_{d} \right\} .\end{eqnarray*}

Filters with convolution kernels of 2, 3 and 4 were constructed to extract key features of Weibo comment text, and then their output vectors were input into the BiLSTM model.

### BiLSTM model

The bi-directional long short-term memory (BiLSTM) model is a variant of the recurrent neural network, which extracts features from the front and back directions to capture the long-distance dependency and context semantic features. This article extracts the emotional features of public opinion event reviews.

**Figure 4:**
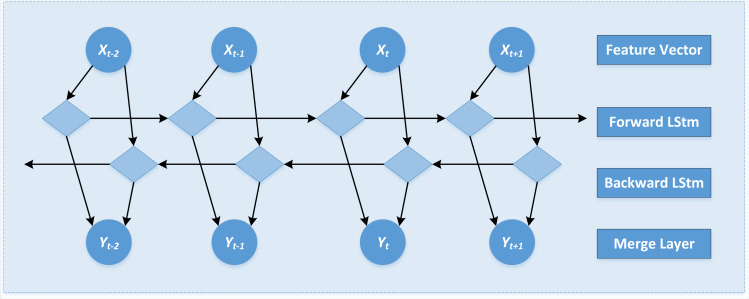
The network structure of the BiLSTM model is shown.

The network structure of the BiLSTM model is shown in Fig. 4,

Through the transmission of state to enhance the subject information and to effectively capture emotional characteristics such as “like” and “haha,” “uncomfortable,” and “pray,” the calculation formulas are shown in Eqs. (4) to (6).

(4) \begin{eqnarray*}\overrightarrow {{h}_{t}}& =f \left( {w}_{1}\times {x}_{t}+{w}_{2}\times \overrightarrow {{h}_{t-1}^{\rightarrow }} \right) \end{eqnarray*}

(5) \begin{eqnarray*}{h}_{t}^{\overleftarrow }& =f \left( {w}_{3}\times {x}_{t}+{w}_{5}\times \overrightarrow {{h}_{t+1}} \right) \end{eqnarray*}

(6) \begin{eqnarray*}{y}_{t}& =g \left( {w}_{4}\times \overrightarrow {{h}_{t}}+{w}_{6}\times {h}_{t}^{\overleftarrow } \right) \end{eqnarray*}

where, ${\vec{h}}_{t}$ represents the state of the forward LSTM layer at time t, and ${h}_{t}^{\overleftarrow }$ $^{\overleftarrow }$ represents the state of the backward LSTM layer at time t. *x*_*t*_ represents the input word vector; *w*_1_ to *w*_6_ represents weight parameters; *f* represents activation function; *y*_*t*_ is the final output of the bidirectional LSTM layer. Finally, the vector obtained by the BiLSTM model is input into the Softmax classifier to realize the emotion classification. That is, the emotion categories of “joy,” “anger,” and “sadness” are predicted.

## Methodology

### Data acquisition

Building a web crawler using Python and XPath technologies to gather comment data on public opinion events on Weibo (https://weibo.com) is the experimental data for this research. 200,000 data sets with emotive color were created after data cleaning and preprocessing. Three data sets—one for each emotion (joy, anger, and sadness) were randomly split into training, test, and validation sets. The ratio of training set, test set and validation set was 3:1:1. Table 1 displays the distribution of the data.

### Evaluation index

A confusion matrix is a standard tool used to evaluate unbalanced data, as shown in Table 1. It can obtain classification accuracy, precision, recall, and F1.

For sentiment analysis of Weibo social media comments, F1 and accuracy are used for experimental evaluation in this article, and the calculation process is shown in Eqs. (7)–(10).

**Table 1:** Distribution of data set.

Category	Training set	Test set	Validation set
Joy	60,000	30,000	10,000
Anger	30,000	10,000	10,000
Sadness	30,000	10,000	10,000
Total	120,000	50,000	30,000

(7) \begin{eqnarray*}\text{Precision}& = \frac{TP}{TP+FP} \end{eqnarray*}

(8) \begin{eqnarray*}\text{Recall}& = \frac{TP}{TP+FN} \end{eqnarray*}

(9) \begin{eqnarray*}{F}_{1}& = \frac{2\times \text{Precision}\times \text{Recall}}{\text{Precision}+\text{Recall}} \end{eqnarray*}

(10) \begin{eqnarray*}\text{Accuracy}& = \frac{TP+TN}{TP+TN+FP+FN} \end{eqnarray*}

Among them, precision is used to evaluate the percentage of emotion classification correctly predicted as the percentage of specified category in the number of anticipated category reviews. The recall is used to assess the percentage of emotion classification correctly predicted in the number of emotion reviews of the category. *F*_1_ is a weighted harmonic mean of precision and recall.

### Experimental process

This article used the LDA model and emotion dictionary. The n_topic of the LDA model was set as three, corresponding to “joy,” “anger,” and “sadness,” respectively. This operation allows unnecessary noise feature words to be effectively filtered, and feature words will be more emotional after processing, which provides good support for the subsequent emotion classification of the CNN-BiLSTM model. The specific process is as follows:

(1) Chinese word segmentation and data cleaning (including stop word filtering and special character cleaning) extract feature words that only retain semantic value information.
(2) The LDA model is used to extract the emotional feature words of “joy,” “anger,” and “sadness,” and the emotional feature words of other comments are added by combining the emotional vocabulary ontology database and manual annotation.
(3) The emotion features are extracted from the CNN-BiLSTM model.

After feature extraction based on the LDA model and emotion dictionary, this article constructs the CNN-BiLSTM model and realizes the social media sentiment analysis experiment. The hyper-parameters of the model are shown in Table 2.

**Table 2:** Hyper-parameters of the CNN-BiLSTM model. The emotion features are extracted from the CNN-BiLSTM model. After feature extraction based on the LDA model and emotion dictionary, this article constructs the CNN-BiLSTM model and realizes the social media sentiment analysis experiment. The hyper-parameters of the model are shown.

Hyper-parameters	Name	Value
L	Length of text sequence	500
d	Word vector dimension	500
m	Filter window size	3,4,5
T_−_cnn	Number of CNN convolution kernels	128
T_−_*l*stm	Number of LSTM hidden layers	128
T_−_*dense*	Number of classifiers	3
lr	Learning rate	0.001
p	Dropout parameter	0.4
Batchsize	Batch gradient drop	25
AF	Activation function	Adam

In addition, the Epoch of the model is 200. A Dropout layer is added to prevent overfitting. To avoid the influence of one abnormal experiment result, the whole experiment result is the average value of ten experiment results. At the same time, it is compared with classical machine learning models (including logistic regression, SVM, random forest, KNN, naive Bayes, AdaBoost) and deep learning models (including LSTM, BiLSTM, Gru, BiGRU, CNN, TextCNN).

## Results and Discussion

### Results of emotional feature word extraction

**Figure 5:**
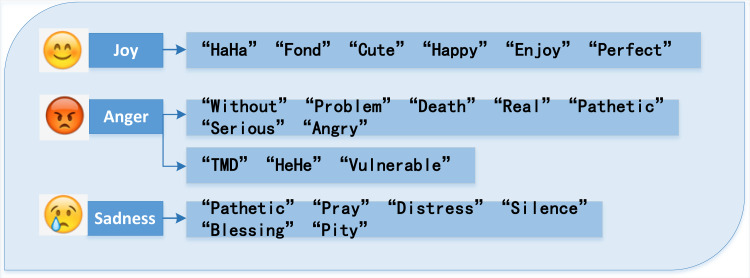
Results of emotional feature words extraction. The emotional feature word extraction model proposed in this article completes the social media sentiment analysis task.

As shown in Fig. 5, the emotional feature word extraction model proposed in this study completes the social media sentiment analysis task. These words are social media lingo and frequently have particular meanings. The “anger” category includes terms like “without,” “problem,” “death,” “real,” “pathetic,” “serious,” and “angry,” as well as Internet terms like “TMD,” “HeHe,” and “vulnerable.” These feature words effectively reflect the public’s anger on public opinion events, and feature extraction based on LDA model and emotion dictionary can effectively enhance the result of eliciting emotion. The key emotional features of the “sadness” category include “pathetic,” “distress,” “silence,” “pray,” “blessing,” and “pity.”

### Comparison of different models

**Figure 6:**
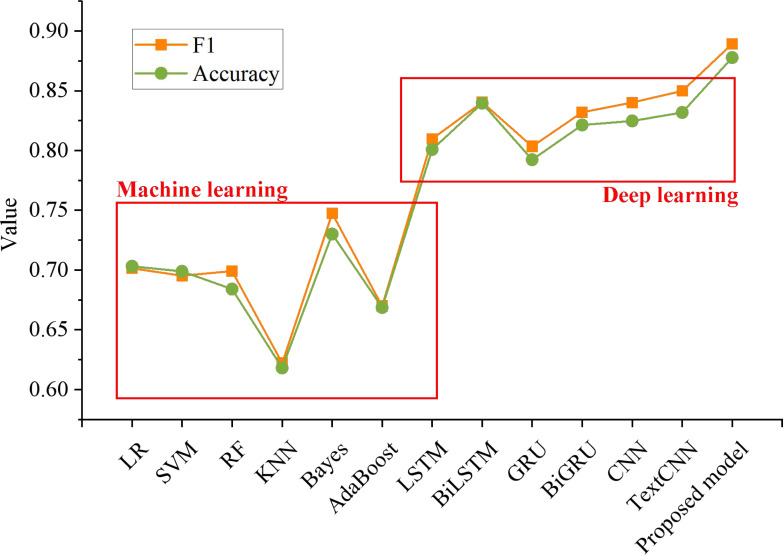
Comparison of emotion classification of different models. The F1 value of the proposed model is 0.89, and the accuracy rate is 0.87. The experimental results are better than the existing machine learning and deep learning models.

Figure 6 shows that the F1 value of the proposed model is 0.89, and the accuracy rate is 0.87. The experimental results are better than the existing machine learning and deep learning models.

By comparing the changing trend of the F1 score between this method and other methods, it can be found that this method is 0.1878, 0.1939, 0.1902, 0.2671, 0.1419 and 0.2194 higher than logistic regression, SVM, random forest, KNN, Naive Bayes and AdaBoost, respectively. In addition, it is 0.0795, 0.0489, 0.0858, 0.0572, 0.0491 and 0.0394 higher than LSTM, BiLSTM, GRU, BiGRU, CNN and TextCNN, respectively. It can be seen that proposed method is 0.1747, 0.1789, 0.1937, 0.2597, 0.1477 and 0.2092 higher than logistic regression, SVM, random forest, KNN, Naive Bayes and AdaBoost, respectively. However, compared with LSTM, BiLSTM, GRU, Bi- GRU, CNN and TextCNN, the accuracy of the proposed model is improved by 0.0769, 0.0382, 0.0855, 0.0564, 0.0530 and 0.0459 respectively.

**Figure 7:**
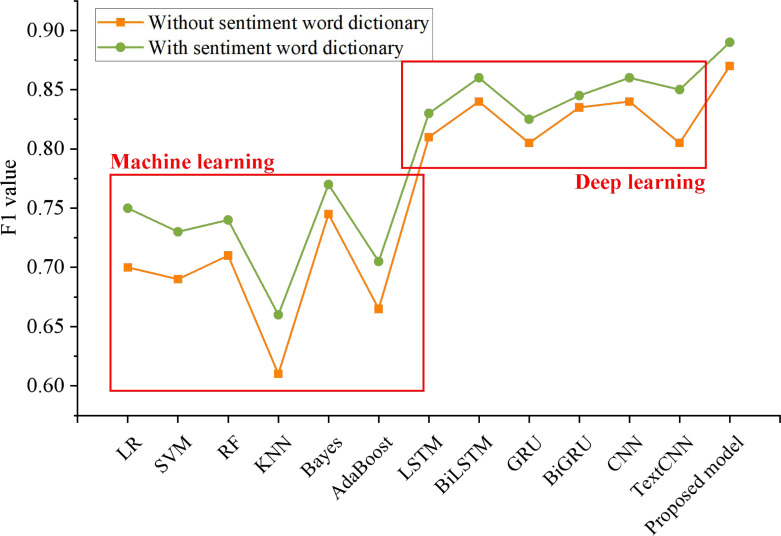
The influence of emotion dictionary on the model. Comparison of the F1 values of the fusion of the LDA model and emotion dictionary in different methods.

In addition, this article compares the F1 values of the fusion of the LDA model and emotion dictionary in different methods, and the experimental results are shown in Fig. 7.

The results show that the fusion of the LDA model and emotion dictionary indicates better performance in sentiment analysis of social media reviews. The F1 value of six machine learning models is increased by 3.66%, and that of seven deep learning models is increased by 1.84%, which shows that the effective extraction of emotional feature words can improve the effectiveness of the classification model to a certain extent. It can fully realize the sentiment analysis of the comments on social media public opinion events, better perceive the public sentiment and predict the emotional trend. It can give full play to the advantages of multilevel and multi-scale feature extraction networks in feature extraction, and can better extract word-level, phrase-level and sentence-level features to ensure the adequacy of feature extraction

### Analysis of different emotional categories

**Figure 8:**
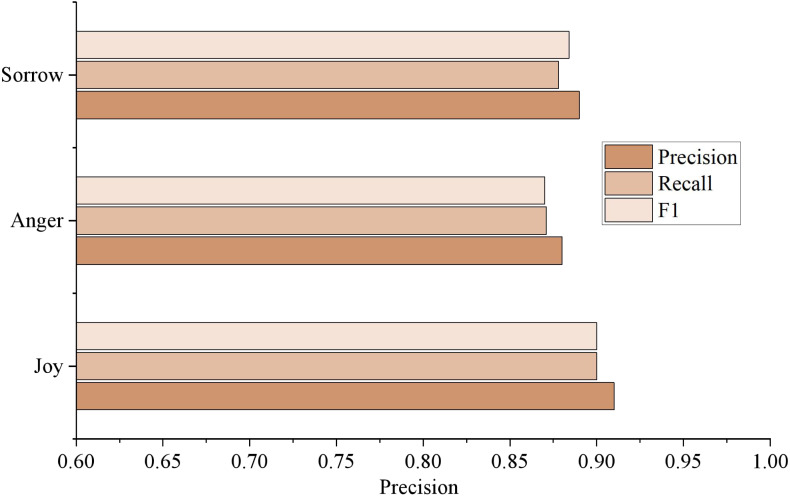
Results of different emotion categories. The analysis results of different emotion categories are shown.

Figure 8 shows the analysis results of varying emotion categories. It can be seen from the figure that the F1 value of “joy” is the highest, which is 0.9062, followed by “sadness” and “anger.” On the one hand, there are a large number of samples of the type of “joy,” and on the other hand, there is a phenomenon of partial integration of the emotional feature words of “anger” and “sadness.” However, the experimental results still effectively prove the method’s effectiveness in this article, which can conduct high-quality emotional trend analysis on the comment information of social media. Automatically distinguishes between different types of emotion such as “joy,” “anger,” and “sadness.”

## Conclusion

This manuscript introduces a model for recognizing the emotions of social media users, which facilitates the emotional analysis of microblog public opinion events. The experimental results reveal the superior performance of our approach, as it yields a precision, recall, F1 value, and accuracy of 0.8946, 0.8841, 0.8893, and 0.8778, respectively, surpassing the existing machine learning and deep learning models. Notably, the LDA model and emotion dictionary’s experimental results significantly improved, with an F1 value 3.66% higher than the six machine learning models and 1.84% higher than the seven deep learning models. In conclusion, our method effectively perceives the emotional situation of public opinion events in social media and holds substantial research value. Nonetheless, this study only uses sorrow, anger, and joy as the indicators of emotion analysis, thereby limiting its scope to coarse-grained analysis. These experimental findings suggest that using CNN alone to extract and learn emotional features is inadequate. In feature extraction, we recommend combining local feature extraction and global feature extraction, emphasizing global feature extraction. Future research should analyze fine-grained emotion characteristics using more complex data annotation.

##  Supplemental Information

10.7717/peerj-cs.1414/supp-1 Supplemental Information 1 Code

## Additional Information and Declarations

### Data Availability

The following information was supplied regarding data availability: The code is available in the Supplemental File. The data is available at Zenodo: tzw. (2023). Emotion recognition of social media [Data set]. Zenodo. https://doi.org/10.5281/zenodo.7622150.
